# Development of Zika Virus Vaccines

**DOI:** 10.3390/vaccines6010007

**Published:** 2018-01-18

**Authors:** Huda Makhluf, Sujan Shresta

**Affiliations:** 1 Department of Mathematics and Natural Sciences, National University, La Jolla, CA 92037, USA; 2 Center for Infectious Disease, La Jolla Institute, La Jolla, CA 92037, USA; sujan@lji.org

**Keywords:** Zika virus, dengue virus, vaccines

## Abstract

Zika virus (ZIKV) is a mosquito-borne flavivirus that emerged as a global threat following the most recent outbreak in Brazil in 2015. ZIKV infection of pregnant women is associated with fetal abnormalities such as microcephaly, and infection of adults can lead to Guillain–Barré syndrome, an autoimmune disease characterized by neurological deficits. Although there are currently licensed vaccines for other flaviviruses, there remains an urgent need for preventative vaccines against ZIKV infection. Herein we describe the current efforts to accelerate the development of ZIKV vaccines using various platforms, including live attenuated virus, inactivated virus, DNA and RNA, viral vectors, and in silico-predicted immunogenic viral epitopes. Many of these approaches have leveraged lessons learned from past experience with Dengue and other flavivirus vaccines.

## 1. Introduction

Zika virus (ZIKV) was originally isolated in 1947 from a febrile rhesus monkey dwelling in the Zika forest canopy in Uganda [1]. The first case of human infection was documented in 1954 in Africa, after which sporadic human infections were recorded. A recent outbreak began in 2007 in the Pacific Yap Islands and was followed by another outbreak in the French Polynesian Islands in 2013, this time with higher rates of neurological disorders [2,3,4]. In 2014, ZIKV entered the Americas, starting in northeastern Brazil and then spreading to many other countries, including the United States [5]. During earlier outbreaks, ZIKV infections were either asymptomatic or presented with mild symptoms resembling those induced by Dengue virus (DENV) infection: a self-limiting febrile illness associated with maculopapular rash, headache, conjunctivitis, and musculoskeletal pain. Since 2014, however, outbreaks in Asia and the Americas have been linked to unusual and severe clinical manifestations, including Guillain–Barré syndrome in adults and congenital ZIKV syndrome (CZS) in infected pregnant women, which has effects ranging from miscarriage to fetal growth retardation, microcephaly, and brain calcifications [6].

## 2. Virology and Disease Pathogenesis

The rapid spread of ZIKV worldwide prompted the World Health Organization (WHO) to declare “Public Health Emergency of International Concern” (February to November 2016). According to the most recent report from the Pan American Health Organization and WHO (early November 2017), the number of ZIKV infections in the Americas since 2015 has reached ~230,000, including 222,986 autochthonous and 6252 imported cases. Notably, there have been 3689 confirmed cases of CZS associated with ZIKV infection [7]. In the United States alone, a total of 225 autochthonous and 5279 imported cases have been reported to date, with 98 confirmed ZIKV-associated CZS cases [7]. Several aspects of ZIKV biology have surprised scientists and clinicians alike. For example, whereas most human flavivirus infections are spread through the bite of infected Aedes aegypti and Aedes albopictus mosquitoes, ZIKV can additionally be transmitted sexually. Indeed, the virus has been documented to persist in semen for up to 6 months [8,9,10]. Between 1 January 2015 and 7 January 2016, at the peak of the epidemic in Brazil, the overall microcephaly birth occurrence increased drastically, with a reported rate of 4.61 per 10,000 live births (32). Pernambuco and Paraíba states confirmed the highest prevalence rates of 14.62 and 10.82 respectively, with approximately a 1:684 ZIKV-related microcephaly births, almost doubling over one year due to ZIKV [11].

ZIKV is an enveloped, positive-stranded RNA virus belonging to the flaviridae family and flavivirus genus. ZIKV and the related DENV share several characteristics because of their phylogenetic closeness and genetic similarity. Both viruses have an ~11 Kb genome encoding a polyprotein that is cleaved into three structural proteins (capsid, premembrane/membrane [prM], and envelope [Env]) and seven nonstructural proteins ([NS1, NS2A, NS2B, NS3, NS4A, NS4B, and NS5]). However, unlike DENV, which has four distinct serotypes, ZIKV exists as a single serotype with two lineages; African and Asian [12].

Although the number of ZIKV cases in the Americas has declined sharply, there remains a dire need for an effective vaccine to prevent further outbreaks. At present, about 45 vaccine candidates are in development and beginning to show promise. Of these, two are currently in phase 2 and nine are in phase 1 clinical trials [13] (Table 1). Here, we describe several of the various platforms for ZIKV vaccines currently in development.

## 3. ZIKV Vaccine Platforms

### 3.1. DNA-Based Vaccines

DNA-based vaccines employ a powerful strategy in which host cells take up viral protein-encoding synthetic DNA and utilize endogenous cellular transcriptional and translational machinery to produce antigenic viral target proteins. DNA-based vaccines are easily engineered, simple to manufacture, and thermostable, allowing easy transport and storage. The vaccines are administered via intradermal electroporation, which enables optimal cellular entry and subsequent protein production. DNA-based vaccines tested to date have been safe and, unlike live attenuated viral vaccines, and are unable to revert and replicate, which is a considerable safety concern, especially in pregnant women.

The DNA-based vaccine candidate VRC ZKADNA085-00-VP (VRC 5288, NCT02840487) was created by the National Institute of Allergy and Infectious Diseases Vaccine Research Center [14,15] and entered phase 1 clinical trials in August 2016. The vaccine design strategy was similar to that employed for vaccines against other flaviviruses, such as Japanese encephalitis virus (JEV) and West Nile virus [16]. VRC5288 comprised full-length prM-Env from ZIKV strain H/PF/2013 together with JEV stem and transmembrane regions, which enhance protein expression and secretion [15]. However, a second candidate virus lacking the JEV modification showed superior immunogenicity and protective efficacy compared with VRC 5288 [15]. This vaccine (VRC-ZKADNA090-00-VP, VRC 5283) contains an optimized DNA construct consisting of unmodified prM-Env and is currently being tested in phase 1 and 2 clinical trials (Table 1; NCT02996461 and NCT03110770, respectively). 

Gaudinski et al. showed that VRC5283 and VRC5288 were well tolerated in healthy adults and produced both cellular and neutralizing antibody responses against ZIKV proteins [17]. Two trials for VRC319 and VRC 320 assessed plasmid VRC5288 and VRC5283 respectively. Gaudinski and colleagues demonstrated that VRC5283 prevented viremia more effectively than VRC5288 while displaying robust neutralizing antibody and T cell responses [17]. The highest immunogenicity was observed in 14 participants receiving VRC5283 at 0, 4, and 8 weeks via split-dose vaccination using a needle-free injection device in each deltoid with a recorded geometric mean titer of 304. As a result, VRC5283 was moved into a phase 2 efficacy trial [17].

A synthetic DNA vaccine, GLS-5700, is in development by GeneOne Life Science/Inovio Pharmaceuticals. GLS-5700 consists of a consensus sequence of prM-Env proteins from infectious ZIKV isolates collected between 1952 and 2015 cloned into the pVax1 vector with an IgE leader sequence to optimize expression [18]. GLS-5700 vaccination of immunocompromised A129 mice, which lack type I (α/β) interferon receptor (Ifnαr1) induced humoral and cellular immunity and 100% protection against weight loss and death following challenge with ZIKV PR209 strain (Puerto Rico) [15]. Furthermore, passive transfer of IgG from GLS-5700-immunized rhesus monkeys protected *Ifnαr1*^−/−^ mice against ZIKV challenge [18]. GLS-5700 is currently being tested in two phase 1 clinical trials to evaluate its immunogenicity, tolerability, and safety in DENV-seropositive and DENV-naïve adults (Table 1, NCT02887482 and NCT02809443). These trials also aim to determine the optimal dose using various delivery regimens. 

Tebas et al. evaluated the immunogenicity and safety of GLS5700 in 40 participants, with a median age of 38 years, divided equally into two groups [19]. The participants were injected with either 1 mg or 2mg of vaccine intra-dermally at baseline, 4 weeks, and 12 weeks later. No adverse events were reported at 14 weeks. 50% of the participants experienced pain at the injection site with either swelling, itching, and/or redness. By week 14, ZIKV specific antibodies developed in all participants. This trial demonstrated the initial safety and immunogenicity of GLS700 [19].

### 3.2. Adenovirus Vector-Based Vaccines

Adenovirus vectors have been widely employed in anti-viral vaccine development and offer several advantages. They are able to induce a broad and robust anti-viral immune response and are easy to manufacture. Adenoviruses from old world monkeys, especially rhesus monkeys, represent a novel class of candidate vaccine vectors as they bypass preexisting immunity to human and chimpanzee adenoviruses [20]. A vaccine based on rhesus adenovirus serotype 52 (RHAd52) prM-Env has been tested in four rhesus monkeys via intramuscular immunization [18]. Notably, a single immunization induced neutralizing antibodies that protected against challenge with ZIKV-BR strain (Brazil, 2015) 4 weeks later [21].

Kim et al. [22] developed a recombinant adenoviral vector, pAd.ZIKV-Efl, that expresses a codon-optimized Env antigen (216–794) from ZIKV strain BeH815744 [22]. To facilitate folding, cleavage, and purification, the Env sequence was linked to the trimerization domain of T4 fibritin (foldon), a Tobacco Etch Virus protease, and a six-histidine tag, respectively. pAd.ZIKV-Efl induced neutralizing humoral immunity in C57BL/6 mice after subcutaneous inoculation with 1011 virus particles followed by a booster shot on day 14. Moreover, ZIKV-specific antibodies were detected as early as 2 weeks after administering a booster shot [22]. Additionally, pups born to mice immunized with pAd.ZIKV-Efl were protected from weight loss and death following a lethal challenge with ZIKV DAKAR41542 strain, confirming passive protection [22].

### 3.3. mRNA-Based Vaccines

Similar to DNA, RNA can be easily synthesized, modified, and incorporated into vaccines. The mRNA-based strategy exploits the host cellular machinery to directly translate mRNA molecules into the viral proteins. Importantly, RNA-based vaccines abrogate the safety risks associated with DNA-based vaccines and potential integration into the human genome.

Pardi et al. [23] developed a nucleoside-modified mRNA vaccine candidate composed of prM-Env from the French Polynesian 2013 ZIKV strain encapsulated in lipid nanoparticles (LNPs), which facilitate biodelivery and presentation. The nucleoside modification (methylpseudouridine) was designed to minimize innate immune system recognition [23]. Inoculation of C57BL/6 and BALB/c mice with this vaccine induced a strong neutralizing antibody response in both mouse strains, and a specific CD4^+^ T cell response was also detected in the BALB/c mice. This vaccine was also tested in rhesus macaques, which were vaccinated and challenged 5 weeks later with Puerto Rico ZIKV strain (PRVABC59). Whereas the placebo-immunized monkeys developed viremia, the mRNA-LNP-vaccinated monkeys were completely protected [23].

Richner and colleagues employed a similar approach to vaccine construction by encapsulating the full-length prM-Env RNA sequence from Micronesia 2017 ZIKV strain into LNPs [24]. To mitigate concerns that the vaccine could induce DENV cross-reactive antibodies, the authors mutated the highly conserved fusion-loop epitope in domain II of the Env protein. The mutant vaccine was confirmed to diminish the production of enhancing anti-DENV antibodies in cells and mice. In AG129 mice, which lack receptors for both type I (α/β) and II (γ) IFNs [24], immunization and boosting with the mutant vaccine induced high titers of neutralizing anti-ZIKV antibodies. When challenged with Malaysia 1966 ZIKV strain P6-740 6 weeks later, the vaccinated AG129 mice survived, whereas the unvaccinated group succumbed to infection. The vaccine also protected immunocompetent C57BL/6 wild-type mice with transient Ifnαr1 blockade. Immunized and boosted mice generated consistently high neutralizing antibody titers and were completely protected from weight loss and death when challenged with ZIKV strain Dakar 41591 13 weeks post-boost [24].

The mRNA platform is versatile and allows various flavivirus wild-type and mutant sequences to be incorporated. In particular, the nucleoside-modified mRNA-LNP vaccines elicit rapid and durable immune protection and represent promising vaccine candidates. Moderna Therapeutics is currently sponsoring a phase 1 clinical trial of their mRNA-1235 ZIKV vaccine to assess its safety, tolerability, and immunogenicity in healthy adults (Table 1, NCT03014089).

### 3.4. Live Attenuated Virus Vaccines

The design of live attenuated ZIKV vaccines has followed a similar strategy to that deployed for the DENV live attenuated tetravalent chimeric vaccine (Dengvaxia), which is licensed on a country by country basis [25,26]. Dengvaxia is a mixture of DENV serotypes 1–4 coupled to NS genes from Yellow fever strain 17D; the Yellow fever vaccine based on these genes has one of the best safety, efficacy, and durability profiles among anti-viral vaccines. In general, the development of live attenuated viruses requires a balance between immunogenicity and safety [25]. On the one hand, a low level of attenuation may promote a robust immune response but cause viremia and neurovirulence. Moreover, there is a possibility of viral reversion, shedding, and transmission. On the other hand, highly attenuated viruses may have better safety profiles, but elicit weakened immune responses. Given that the WHO has assigned the highest vaccination priority to women of child-bearing age, it seems prudent to avoid using live attenuated ZIKV vaccines in this population as well as in immunocompromised individuals [27].

Shan et al. created a live attenuated ZIKV vaccine by deleting 10 nucleotides in the 3’-UTR region of the ZIKV genome [28]. They demonstrated that the vaccine is immunogenic and protective in both immunocompromised AG129 mice and immunocompetent CD-1 mice. Additionally, 1-day-old CD-1 pups survived an injection of 1 × 104 infectious focus units of the 10-del-ZIKV, whereas a dose of only 10 infectious focal units of wild-type ZIKV was lethal. The reduced virulence of the vaccine may have been due to increased sensitivity to the effects of type I IFN and diminished synthesis of viral RNA [28]. This vaccine candidate merits further development and investigation.

Xie et al. constructed two chimeric viruses encoding heterologous (ZIKV and DENV serotype 2) prM-Env structural genes [29]. Chimeric virus I replaced ZIKV prM-Env with DENV2 prM-Env in the backbone of the ZIKV genome. Chimeric virus II consisted of the infectious backbone of DENV2 in which the sequence encoding the 14 C-terminal amino acids from DENV2 prM-Env was substituted with the sequence encoding the 18 C-terminal amino acids of prM-Env from ZIKV strain FSS13025. Both viruses had attenuated virulence and were immunogenic in A129 *Ifn*α*r1*^−/−^ mice. Moreover, the I and II vaccines induced highly protective immunity following DENV2 and ZIKV challenge, respectively [29].

Despite the promise of live attenuated vaccines, they carry myriad safety issues in pregnant women, suggesting that they might best be deployed to protect adolescents and children prior to sexual maturity and activity. In April 2017, Themis Bioscience (Vienna, Austria) launched the first worldwide study of a live attenuated ZIKV vaccine produced by adaptation of their proprietary technology, which utilizes a measles virus backbone (Table 1, NCT03014089). 

### 3.5. Purified Inactivated Virus Vaccines

Unlike vaccines composed of live attenuated viruses, inactivated vaccines negate the possibility of reactivation and replication and are thus not contraindicated in pregnant women or immunocompromised individuals. However, the attenuation also means that more immunization/boosting strategies may be required to ensure long-term protection. The need for an adjuvant might be required, but could complicate the use of a purified inactivated virus (PIV) in pregnancy.

The US Army tested a PIV vaccine based on the Puerto Rican isolate PRVABC59, which was inactivated with 0.05% formalin, purified, and formulated with alum for administration. A single immunization of BALB/c mice with the vaccine conferred protection against viremia upon challenge with ZIKV-BR strain 4 weeks later. This ZIKV PIV vaccine was also tested for immunogenicity and protective efficacy in rhesus monkeys. Consistent with the mouse study, the vaccine induced ZIKV-specific neutralizing antibodies and protection against subsequent challenge with ZIKV-BR and ZIKV-PR strains. Virus was undetectable in blood, urine, or cerebrospinal fluid of the vaccinated monkeys [21,30]. Additionally, adoptive transfer of purified IgG from vaccinated mice conferred passive protection.

ZIKV PIV vaccines are currently being tested in four phase 1 clinical trials sponsored by the National Institute of Allergy and Infectious Diseases and Beth Israel Deaconess Medical Center. The trials are designed to evaluate their immunogenicity, reactogenicity, and safety in flavivirus-primed and -naïve healthy subjects (Table 1; NCT02963909, NCT02952833, NCT03008122, and NCT02937233). Modjarrad showed that a ZPIV candidate vaccine elicited robust neutralizing antibody titers in healthy human participants [31]. 55 healthy adults received two intramuscular injections of 5 μg of ZPIV with aluminum hydroxide adjuvant and 12 participants a placebo injection [31]. 92% (52 out of 55) of these participants seroconverted by day 57 while no placebo participants changed titers. ZPIV was well tolerated, resulting in mild to moderate adverse effects such as pain at the injection site, malaise, headache, and fatigue [31]. 

### 3.6. In Silico Approaches to Vaccine Design

In addition to these “classical” strategies for vaccine development, scientists are exploring immune-informatics approaches to optimize the design of epitope-based vaccines. Using in silico predictive algorithms, Dikhit et al. [32] identified nine HLA class I-restricted ZIKV epitopes that elicited responses from human CD8^+^ T cells. The epitopes mapped to sequences conserved across all known ZIKV strains in five proteins: capsid (MVLAILAFL), envelope (RLKGVSYSL and RLITANPVI), NS2A (AILAALTPL), NS4B (LLVAHYMYL and LVAHYMYLI), and NS5 (SLINGVVRL, ALNTFTNLV and YLSTQVRYL). Collectively, these nine peptides are capable of binding to at least one HLA molecule from 99% of the global population, regardless of ethnicity [32].

While the in silico approach to ZIKV vaccine design clearly holds great promise, the precise immunological significance and relevance of the identified epitopes will require further investigation and validation in cell culture and animal models.

## 4. The Importance of T Cells in Vaccine Design

All ZIKV vaccine candidates currently in clinical trials are designed to elicit neutralizing antibodies as the primary mechanism of protection. However, emerging literature indicates an important role for T cells in fighting ZIKV infection [33,34,35,36], suggesting that an ideal vaccine would trigger both cellular and humoral immune responses. In particular, CD8^+^ T cells have been reported to play an important role in controlling ZIKV infection in various mouse models, including *LysMCre+ Ifn*α*r1^fl/fl^* mice, which lack the IFN α/β receptor in a subset of myeloid cells, and thus have Ifnαr1-competent T cell responses [29], HLA transgenic *Ifn*α*r1*^−/−^ mice [35], and wild-type mice [36]. Additionally, Wen et al. investigated whether CD8^+^ T cell immunity generated during primary DENV infection can confer protection against secondary ZIKV infection in mice [35]. These authors found that DENV-immune *Ifn*α*r1*^−/−^ or DENV-immune wild-type C57BL/6 mice have cross-reactive immunity to subsequent ZIKV infection, which is predominantly mediated by DENV-immune CD8^+^ T cells. Both serotype-specific and cross-reactive antibody responses are generated during primary DENV infection [37]. Secondary DENV infection results in severe blood vessel leakage and hemorrhagic fever. This is partly due to the ability of cross-reactive antibodies to induce the formation of complexes with DENV which can effectively bind Fcγ receptors [37], a process referred to as antibody dependent enhancement, which does not occur in the vast majority of secondary infections. Bardina et al. investigated whether antibody dependent enhancement applied to ZIKV infection and found that administration of West Nile Virus and DENV plasma into ZIKV susceptible mice caused a surge in mortality and morbidity [38]. Hence, DENV vaccines that elicit a T cell response may confer cross-protection against ZIKV infection without complications from antibody-dependent enhancement [34,35]. Yang et al. showed that a virus-like particle carrier based on the hepatitis B core antigen, which displays ZIKV envelope protein domain III, induced potent neutralizing immune responses in mice [39]. Moreover, this devised vaccine induced neutralizing immunity while circumventing the induction of antibodies with ADE activity for DENV infections [39]. Yet another alternative vaccine strategy was developed by Brault and colleagues [40] to avert the potential risk of ADE associated with structural ZIKV proteins [40]. The NS1 gene of a 2015 Asian isolate was inserted into a modified vaccinia Ankara (MVA) vector to generate MVA-ZIKV-NS1 vaccine. Immunization of immunocompetent mice resulted in both a robust cellular and humoral response as well as 100% protection against a lethal intracerebral dose of MR766 ZIKV strain [40]. 

A critically important research objective will be a comprehensive investigation of ZIKV vaccine-associated immunologically-enhanced disease in humans to determine the underlying immune-pathologic mechanisms of severe and atypical ZIKV disease. 

## 5. Conclusions

Regardless of the platform, all ZIKV vaccine candidates must undergo clinical testing to optimize dose, schedule, and delivery route, as well as to evaluate vaccine immunogenicity, efficacy, and safety. These studies may be hindered by the decline in ZIKV infections worldwide, which most likely stems from herd immunity. Nevertheless, the potential for future outbreaks is unknown, and the development of an effective vaccine must remain a priority. Adaptation of the existing flavivirus vaccine platforms appears promising. However, we must remain attuned to the possibility that prior exposure to flaviviruses may worsen ZIKV infections, a possibility that certainly warrants further investigation. Another important safety concern is the potential for ZIKV vaccines to elicit Guillain–Barré syndrome complications in adults.

The beginning of the 21st century has ushered in an era of personalized medicine. Innovative technologies in genomics and bioinformatics platforms are changing the way we approach vaccine design. It may soon be possible to consider “precision vaccination”, in which an individual’s HLA type or genetic predisposition to side effects is taken into account during vaccine design.

In conclusion, the development of a prophylactic vaccine for the populations most vulnerable to ZIKV infection—pregnant women and babies—should remain a priority for scientists and clinicians alike. ZIKV and other pathogens known to cause congenital infection and disease such as cytomegalovirus remain to be overcome. History shows that successful collaboration between basic and clinical researchers can overcome the devastating effects of in utero viral infections, as illustrated by the virtual eradication of congenital rubella syndrome once the rubella vaccine was added to standard pediatric immunization programs. 

## Figures and Tables

**Table 1:** ZIKV vaccine candidates in clinical trials.

Vaccine Candidate	Sponsor	Platform	Trial Registry ID	Immunogen	Phase	Status
VRC-ZKADNA085-00-VP	NIAID	DNA	NCT02840487	prME	1	Ongoing, not recruiting
VRC-ZKADNA090-00-VP	NIAID	DNA	NCT02996461	prME	1	Ongoing, not recruiting
VRC-ZKADNA090-00-VP	NIAID	DNA	NCT03110770	prME	2	Open, recruiting
GLS-5700	GeneOne Life Science/Inovio Pharmaceuticals	DNA	NCT02809443	prME	1	Ongoing, not recruiting
GLS-5700	GeneOne Life Science/Inovio Pharmaceuticals	DNA	NCT02887482	prME	1	Ongoing, not recruiting
MV-Zika	Themis Bioscience	Recombinant viral vector	NCT02996890	prME	1	Ongoing, not recruiting
mRNA-1325	Moderna Therapeutics	mRNA	NCT03014089	prME	2	Open, recruiting
ZIKV PIV	NIAID	Inactivated whole target organism	NCT02963909	whole virus	1	Ongoing, not recruiting
ZIKV PIV	NIAID	Inactivated whole target organism	NCT02952833	whole virus	1	Open, recruiting
ZIKV PIV	BIDMC	Inactivated whole target organism	NCT02937233	whole virus	1	Ongoing, not recruiting
ZIKV PIV	NIAID	Inactivated whole target organism	NCT03008122	whole virus	1	Open, recruiting

BIDMC: Beth Israel Deaconess Medical Center; NIAID: National Institute of Allergy and Infectious Diseases; PIV: purified inactivated vaccine; prME: premembrane and envelope proteins.
